# Estimating COVID-19 Pneumonia Extent and Severity From Chest Computed Tomography

**DOI:** 10.3389/fphys.2021.617657

**Published:** 2021-02-15

**Authors:** Alysson Roncally Silva Carvalho, Alan Guimarães, Thiego de Souza Oliveira Garcia, Gabriel Madeira Werberich, Victor Fraga Ceotto, Fernando Augusto Bozza, Rosana Souza Rodrigues, Joana Sofia F. Pinto, Willian Rebouças Schmitt, Walter Araujo Zin, Manuela França

**Affiliations:** ^1^Cardiovascular R&D Centre (UnIC), Department of Surgery and Physiology, Faculty of Medicine, University of Porto, Porto, Portugal; ^2^Laboratory of Pulmonary Engineering, Biomedical Engineering Program, Alberto Luiz Coimbra Institute of Post-Graduation and Research in Engineering, Universidade Federal do Rio de Janeiro, Rio de Janeiro, Brazil; ^3^Laboratory of Respiration Physiology, Carlos Chagas Filho Institute of Biophysics, Universidade Federal do Rio de Janeiro, Rio de Janeiro, Brazil; ^4^Medical Faculty, Universidade Estácio de Sá, Rio de Janeiro, Brazil; ^5^Department of Radiology, Universidade Federal do Rio de Janeiro, Rio de Janeiro, Brazil; ^6^Hospital Niteroi D’Or, Niterói, Brazil; ^7^D’Or Institute for Research and Education, Rio de Janeiro, Brazil; ^8^National Institute of Infectious Disease, Oswaldo Cruz Foundation, Rio de Janeiro, Brazil; ^9^Radiology Department, Complexo Hospitalar Universitário do Porto, Porto, Portugal; ^10^Instituto de Ciências Biomeìdicas Abel Salazar, Porto University, Porto, Portugal

**Keywords:** computed tomography, COVID-19, deep learning, CT-estimated lung volume, CT-estimated lung weight

## Abstract

**Background:**

COVID-19 pneumonia extension is assessed by computed tomography (CT) with the ratio between the volume of abnormal pulmonary opacities (PO) and CT-estimated lung volume (CT_LV_). CT-estimated lung weight (CT_LW_) also correlates with pneumonia severity. However, both CT_LV_ and CT_LW_ depend on demographic and anthropometric variables.

**Purposes:**

To estimate the extent and severity of COVID-19 pneumonia adjusting the volume and weight of abnormal PO to the predicted CT_LV_ (pCT_LV_) and CT_LW_ (pCT_LW_), respectively, and to evaluate their possible association with clinical and radiological outcomes.

**Methods:**

Chest CT from 103 COVID-19 and 86 healthy subjects were examined retrospectively. In controls, predictive equations for estimating pCT_LV_ and pCT_LW_ were assessed. COVID-19 pneumonia extent and severity were then defined as the ratio between the volume and the weight of abnormal PO expressed as a percentage of the pCT_LV_ and pCT_LW_, respectively. A ROC analysis was used to test differential diagnosis ability of the proposed method in COVID-19 and controls. The degree of pneumonia extent and severity was assessed with Z-scores relative to the average volume and weight of PO in controls. Accordingly, COVID-19 patients were classified as with limited, moderate and diffuse pneumonia extent and as with mild, moderate and severe pneumonia severity.

**Results:**

In controls, CT_LV_ could be predicted by sex and height (adjusted *R*^2^ = 0.57; *P* < 0.001) while CT_LW_ by age, sex, and height (adjusted *R*^2^ = 0.6; *P* < 0.001). The cutoff of 20% (AUC = 0.91, 95%CI 0.88–0.93) for pneumonia extent and of 50% (AUC = 0.91, 95%CI 0.89–0.92) for pneumonia severity were obtained. Pneumonia extent were better correlated when expressed as a percentage of the pCT_LV_ and pCT_LW_ (*r* = 0.85, *P* < 0.001), respectively. COVID-19 patients with diffuse and severe pneumonia at admission presented significantly higher CRP concentration, intra-hospital mortality, ICU stay and ventilatory support necessity, than those with moderate and limited/mild pneumonia. Moreover, pneumonia severity, but not extent, was positively and moderately correlated with age (*r* = 0.46) and CRP concentration (*r* = 0.44).

**Conclusion:**

The proposed estimation of COVID-19 pneumonia extent and severity might be useful for clinical and radiological patient stratification.

## Key points

- COVID-19 pneumonia extent and severity can be less biased computed when adjusted to the predicted CT_LV_ and CT_LW_, respectively.
- COVID-19 patients classified as having diffuse and more severe pneumonia had worse clinical and radiological outcomes.
- COVID-19 pneumonia extent better correlates with its severity when both were adjusted to the predicted CT_LV_ and CT_LW_.

## Introduction

Chest computed tomography (CT) has been used widely to assess 2019 coronavirus disease (COVID-19) pneumonia and is key for the detection of abnormal parenchymal opacities (PO) and the evaluation of disease extent and severity (Hope et al., 2020). The extent of COVID-19 pneumonia is often determined on chest CT images by computing the volume of abnormal PO adjusted to the CT–estimated lung volume (CT_*L*__*V*_) (Colombi et al., 2020; Yang et al., 2020).

During pneumonia progression, regardless of its etiology, total lung volume is reduced (Patroniti et al., 2005). However, total lung weight can slightly increase in less severe pneumonia, while a significant increase in the lung weight is expected in more severe pneumonia. For instance, if a CT scan exhibits PO, but the total lung weight is in the normal range, atelectasis would be a likely explanation for this parenchymal opacity (Brismar et al., 1985). However, if a similar PO is associated with an increase in lung weight, consolidation resulting from a more relevant lung injury due to hemorrhage, contusion, or edema from capillary leakage might be raised (Puybasset et al., 2000a; Patroniti et al., 2005; Gattinoni et al., 2006). Thus, lung volume reduction at the expense of alveolar flooding by edema/hemorrhage seems to imply a more pronounced increase in lung weight, which could be a marker of pneumonia severity (Reske et al., 2011).

In fact, CT–estimated lung weight (CT_LW_) has also been used for assessing COVID-19 pneumonia severity; greater pneumonia severity is likely associated with greater elastance and intrapulmonary shunting, and CT_LW_ as great as 1.5 kg have been reported (Gattinoni et al., 2020; Rello et al., 2020) in more severe COVID-19 patients.

Despite the CT_LV_ and CT_LW_ are associated with pneumonia extent and severity, both also depend on anthropometric and demographic variables; they should be greater in males than in females and increase with subject height (Whimster and Macfarlane, 1974; Mull, 1984; Cressoni et al., 2013; Protti et al., 2014). Accordingly, CT_LV_ and CT_LW_ dependencies on demographic and anthropometric variables likely influence the estimation of disease extent and severity.

To overcome this limitation, we proposed that pneumonia extent should be computed from chest CT-estimates volume of abnormal PO adjusted to the predicted lung volume (pCT_LV_). Similarly, pneumonia severity might be estimated from CT-estimates weight of the same abnormal PO adjusted to the predicted CT-estimates lung weight (pCT_LW_). This approach involves applying predictive equations for the extrapolation of total lung volume and tissue contents to establish a threshold to assess the extent and severity of COVID-19 pneumonia. We hypothesize that this approach would prove helpful in clinical practice by yielding a less biased non-invasive diagnostic indicator of patient risk stratification.

## Materials and Methods

### COVID-19 Patients

This retrospective study was conducted with 103 consecutive patients with COVID-19 pneumonia (69 males, 34 females), confirmed by reverse-transcription polymerase chain reaction of nasopharyngeal swab samples, who were admitted to three hospitals (Hospital Copa Star and Barra D’Or, Rio de Janeiro, Brazil and Hospital de Santo António, CHUP, Porto, Portugal) in April–July 2020 and underwent chest CT examination, and for whom demographic and anthropometric data were available.

### Control Subjects

Chest CT images from 86 subjects (24 males, 62 females) that underwent a helical chest CT scan for clinical purposes, and whose images were considered non-pathological by radiologists, were retrospectively included in this study. Exclusion criteria were age <18 years; use of contrast agents; situs inversus; previous pulmonary lobectomy/segmentectomy; and pulmonary diseases (as pneumonia, interstitial pneumopathies, pulmonary fibrosis, emphysema, chronic obstructive pulmonary disease, pulmonary tuberculosis, ARDS, lung nodules, lung cancer, pneumothorax, pleural effusion, mesothelioma).

The hospitals’ research ethics committees approved the study, which complied with current national and international standards (CHUP, 075-DEFI/076-CE; IDO’r, CAAE 29496920.8.0000.5262). Since the study was retrospective, the institutional review board of all hospitals waived the necessity for collecting informed consent from patients.

**FIGURE 1:**
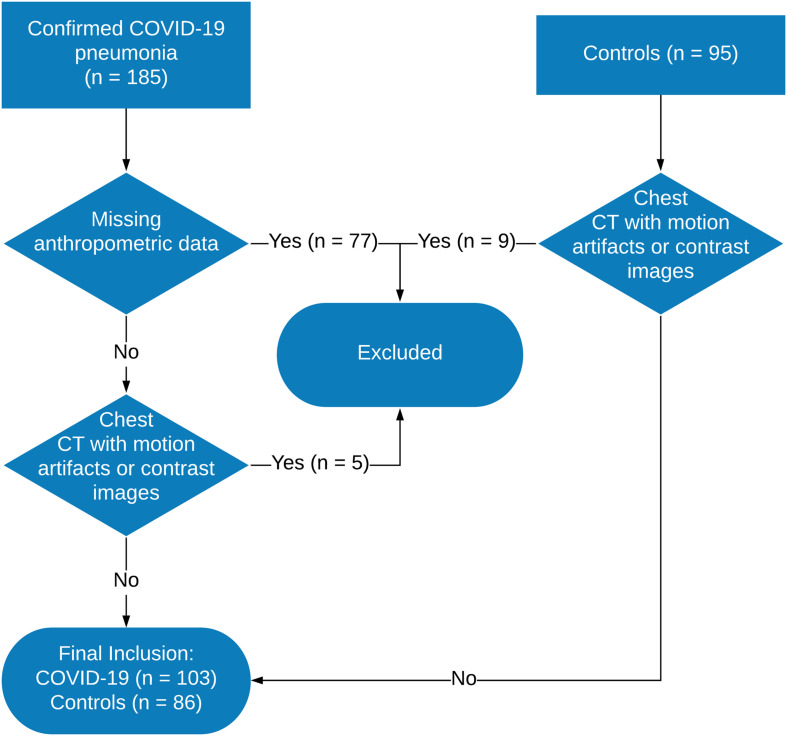
Flowchart of patient enrollment and computed tomography image selection. CT, computed tomography; COVID-19, 2019 coronavirus disease; RT-PCR, reverse-transcription polymerase chain reaction.

The collection of morphometric data occurred in two phases: sex and age were recorded before CT execution, while height and weight were declared later by each subject, without the possibility to directly measure them. Figure 1 shows the flow of subjects’ enrollment and CT scan selection.

### Chest Computed Tomography Acquisition

CT scans were performed on a 64-channel multislice (Brilliance 40 scanner, Philips Medical Systems, Cleveland, OH, United States; and General Electrics Lightspeed VCT, Chicago Illinois, United States), a 128-channel multislice dual-source CT system (Somatom Definition Flash, Siemens, Forchheim, Germany), or a 16-channel multislice (Emotion 16 CT, Siemens, Erlangen, Germany). The acquisitions were gathered with patients in the supine position at end-inspiratory holds, with 120 kV and 120–300 mA, slice thickness ranging from 1 to 2 mm with 50% superposition, and 512 × 512, 768 × 768, or 1,024 × 1,024 voxels matrix. Reconstruction algorithms were C(1), FC13(5), FC86(1), L(79), B50f(35), B60f(1), B70s(12), I50f/2(1), LUNG(51), SOFT(3), depending on the CT manufacture.

### Image Processing and CT_LV_ and CT_LW_ Calculation

The lung parenchyma and airways were segmented from the chest CT images (Fedorov et al., 2012), and the resulting images were exported to an in-house program (Quantitative Lung Image, QUALI) written in MATLAB^®^ (MathWorks^®^, Natick, MA, United States). The images were rescaled for comparison across cases as described previously (Staring et al., 2016).

The CT_LV_ (sum of air and tissue volumes) was calculated as:

(1) $${\text{CT}}_{\text{LV}}(\text{mL})\;=\;{\text{pixel size}}^{2}\times \text{ slice thickness }\times \text{ total number of pixels for the whole lung.}$$

The CT_LW_ was calculated as:

(2) $${\text{CT}}_{\text{LW}}(\text{g})\;=\;[(\text{HU}\;\text{–}\;{\text{HU}}_{\text{Air}}){\text{/(HU}}_{\text{Aorta}}\;\text{—}\;{\text{HU}}_{\text{Air}})]\;\times \;\text{voxel volume}\;\times \;1.04\;\text{g/mL,}$$

where 1.04 mg/mL is the lung tissue density and HU is the voxel density on the HU scale (Staring et al., 2016).

### CT_LV_ and CT_LW_ Prediction

The pCT_*L*__*V*_ and pCT_LW_ were calculated using images from the control group and a multiple linear regression model, taking subjects’ age, sex, and height as initial predictors in accordance with Eqs 3 and 4, respectively:

(3) $${\text{pCT}}_{\text{LV}}\left(\text{mL}\right)=\text{A}\times \text{age}\left(\text{y}\right)+\text{B}\times \text{sex}+\text{C}\times \text{height}\left(\text{m}\right)+\text{D}$$

and

(4) $${\text{pCT}}_{\text{LW}}\left(\text{g}\right)={\text{A}}^{\prime }\times \text{age}\left(\text{y}\right)+{\text{B}}^{\prime }\times \text{sex}+{\text{C}}^{\prime }\times \text{height}\left(\text{m}\right)+{\text{D}}^{\prime }$$

where A, A′, B, B′, C, C′ and D, D′ are the coefficients to be determined. Adjustment for sex was performed by including a dummy-coded sex variable (male = 0).

### Visual Classification of Radiological Patterns in COVID-19 CT Scans

Two chest radiologists blinded to patient identification, clinical data, and outcomes, independently selected up to four ROIs per COVID-19 patient visually classified as well-aerated, ground-glass opacities (GGO), crazy paving/linear opacities (CP/LOs), and consolidation. The ROI consisted of a circle with a fixed radius of 4 mm with a spanning area of about 30 voxels in each CT section (Carvalho et al., 2020).

### Development of the Supervised Neural Network Architecture

A complete description of the supervised artificial neural network (ANN) development was described in Carvalho et al. (2020). Briefly, a density histogram calculated from ROIs and the respective quantiles (2.5, 25, 50, 75, and 97.5%) were used to train a supervised ANN. The unweighted Cohen’s kappa test between the ANN classification and their respective ROI classification attributed by the radiologists was used to assess ANN classifier.

Detailed ANN performance can also be obtained in Carvalho et al. (2020). To determine the degree of PI, the ANN identified abnormal parenchymal opacities and two radiologists blinded to patient identification, clinical data, and outcomes independently validated the results.

### Determination of Pneumonia Extent in COVID-19

Pneumonia extent was calculated as the cumulative volumetric sum of GGO, CP/LOs, and consolidation, also referred as abnormal PO, adjusted to the pCT_LV_.

A receiver operating characteristic (ROC) curve was used to test the differential diagnosis ability of the volume of PO, expressed as a percentage of the pCT_LV_, in controls and COVID-19 patients. The area under the ROC curve (AUC) was assessed and the threshold sensitivity, specificity, accuracy, positive and negative predictive values, *F*-score, and Matthews correlation coefficient were computed.

To evaluate the degree of pneumonia extent in patients with COVID-19, we used the *Z*-score in relation to the average volume of lung PO in the control group adjusted to the pCT_LV_ and expressed as standard deviation units. *Z*-scores for patients with COVID-19 that exceeded the control values were deemed positive and those below this value were deemed negative. Then, patients with COVID-19 were classified as having limited (pneumonia extent < ROC threshold), moderate (ROC threshold ≤ pneumonia extent < *Z*-score 3), or extensive (pneumonia extent ≥ *Z* score 3) pneumonia.

### Determination of Pneumonia Severity in COVID-19

Pneumonia severity was calculated as the weight of the same PO identified for pneumonia extent quantification adjusted to the pCT_LW_.

The same procedure described above for computation of pneumonia extent was adopted to determine the threshold of normality and as well as the degree of the pneumonia severity in COVID-19 patients. Thus, patients with COVID-19 were classified as presenting mild (pneumonia severity < ROC threshold), moderate (ROC threshold ≤ pneumonia severity < *Z* score 3), or severe (pneumonia severity ≥ *Z* score 3) pneumonia severity.

### Clinical and Laboratory Data, Definitions, and Outcomes

Clinical and laboratory findings of each patient were recorded at admission. CT was performed within 12 h after the clinical evaluation and laboratory findings.

Serum C-reactive protein concentration (CRP) collected at the admission was used as a marker of systemic inflammation. ICU stay, as well as ventilatory support, and the intra-hospital mortality were assumed as clinical outcomes. The volume and weight of consolidation, CP/LO, and ground glass opacities were computed by the ANN and used as radiological outcomes.

### Statistical Analysis

The normality of the data was examined using the Kolmogorov–Smirnov test with Lilliefors’ correction, and the homogeneity of variance was assessed using the Levene median test. As both conditions were satisfied, all data are presented as means and standard deviations.

The relationship between patient’s height, weight, age, sex and CT_LV_ and CT_LW_ was assessed by multiple linear regression. The Bland-Altman graphic method was used to evaluate the concordance between the measured and predicted CT_LV_ and CT_LW_ in controls and COVID-19 patients.

A student *t*-test was used to compare measured and predicted CT_LV_ and CT_LW_ in controls and COVID-19 subjects. The one-way ANOVA test followed by Bonferroni *post hoc* test was used to assess statistical differences among patients with limited, moderate, and diffuse pneumonia extent, as well as among those with mild, moderate, and severe COVID-19 pneumonia severity, and control subjects.

The correlation of pneumonia extent and severity as well as between pneumonia extent and severity with clinical and demographic data was assessed using Pearson correlation analysis (very weak, *r* = 0.00–0.19; weak, *r* = 0.20–0.39; moderate, *r* = 0.40–0.59; strong, *r* = 0.60–0.79; very strong, *r* ≥ 0.80). *P* < 0.05 were considered to be significant. All statistical analyses were performed using MATLAB^®^ software (MathWorks^®^).

## Results

The ANN architecture with a single hidden layer of 60 neurons showed the best agreement in the confusion test matrix among the other architectures tested with an overall agreement of 86% being 100% for well-aerated, 76% for GGO, 72% for CP/LO, and 100% for consolidation.

No improvement in the performance of the ANN classifier was observed with the addition of a second neuron layer. The classifier performance was much better for well-aerated and consolidation with an AUC of 1.00 and 0.99. The performance for GGO and CP/LO, despite lower, was quite acceptable with an AUC of 0.94 and 0.91, respectively. The best validation performance occurred at epoch 6.

**FIGURE 2:**
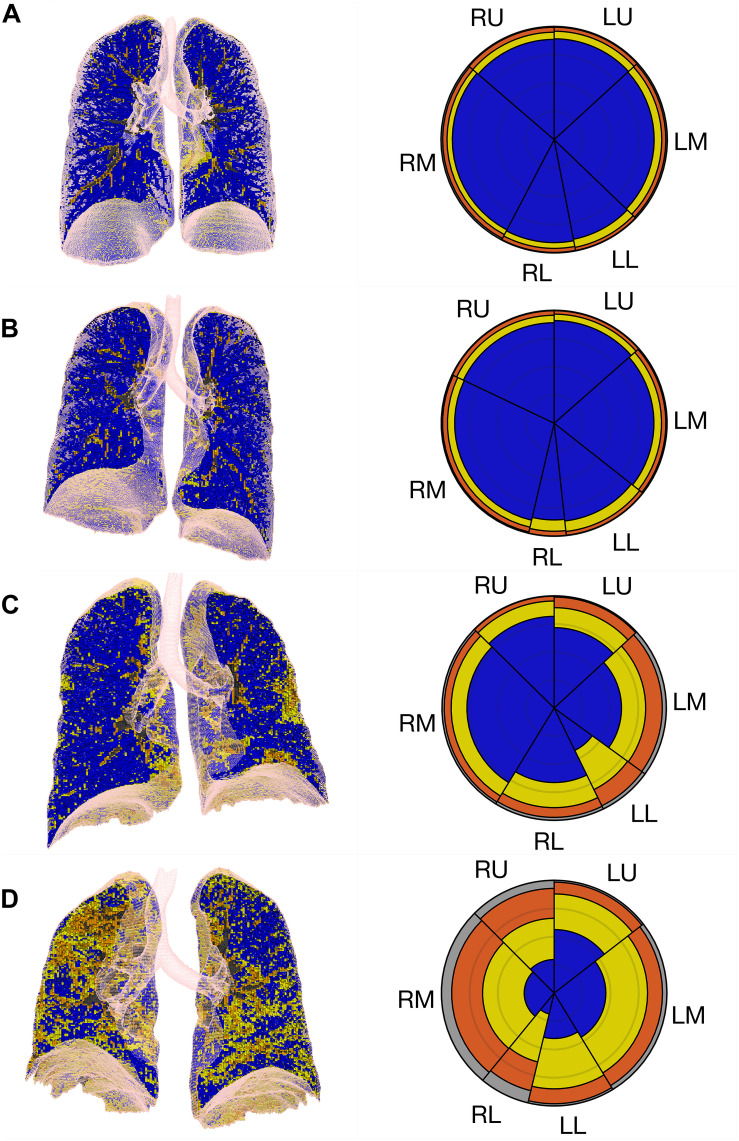
Summary glyphs (right column) derived from three-dimensional imaging data (left column) for a representative control case (row **A**) and cases of COVID-19 with limited and mild (row **B**), moderate (row **C**), and diffuse and severe (row **D**) pneumonia extent and severity. R, right lung; L, left lung; U, upper lung; M, middle lung; L, lower lung. Ground-glass opacities (GGO) are colored in yellow, crazy-paving/reticular opacities in orange and consolidation in gray.

In control subjects, most ANN-classified parenchymal opacities represented small bronchi, peribronchial vessels, and pleural or diaphragm interfaces likely related to partial volume effects and were color-coded as yellow, orange and gray (Figure 2A). In COVID-19 patients (Figures 2B–D), parenchymal opacities, were therefore interpreted, in addition to the peribronchial vessels, as GGO (yellow), CP/LO (orange) and consolidation (gray) broadly spread over the lung parenchyma in COVID-19 with disuse pneumonia extent (Figure 2D).

Lung volume was related significantly to sex (*P* = 0.0002) and height (*P* < 0.0001), but not age. Thus, the pCT_LV_ (*R*^2^ = 0.57, adjusted *R*^2^ = 0.56, F-statistic = 55.5 and *P* < 0.0001) could be predicted as:

(5) $${\text{pCT}}_{\text{LV}}\left(\text{mL}\right)=4808.1\times \text{height}\left(\text{m}\right)-3602.5$$

For males, 800.6 mL should be added to the pCT_LV_. A residual standard error of 634.3 mL on 83 degrees of freedom was obtained with this regression model.

Lung weight was related significantly to age (*P* = 0.011), sex (*P* = 0.015), and height (*P* < 0.0001; *R*^2^ = 0.60, adjusted *R*^2^ = 0.58, *F*-statistic = 40.6 and *P* < 0.0001), being calculated as:

(6) $${\text{pCT}}_{\text{LW}}\left(\text{g}\right)=-1.8\times \text{age}+795.2\times \text{height}\left(\text{m}\right)-573.8$$

For males, 83 g should be added to the pCT_LW_. A residual standard error of 103.8 g on 82 degrees of freedom.

**FIGURE 3:**
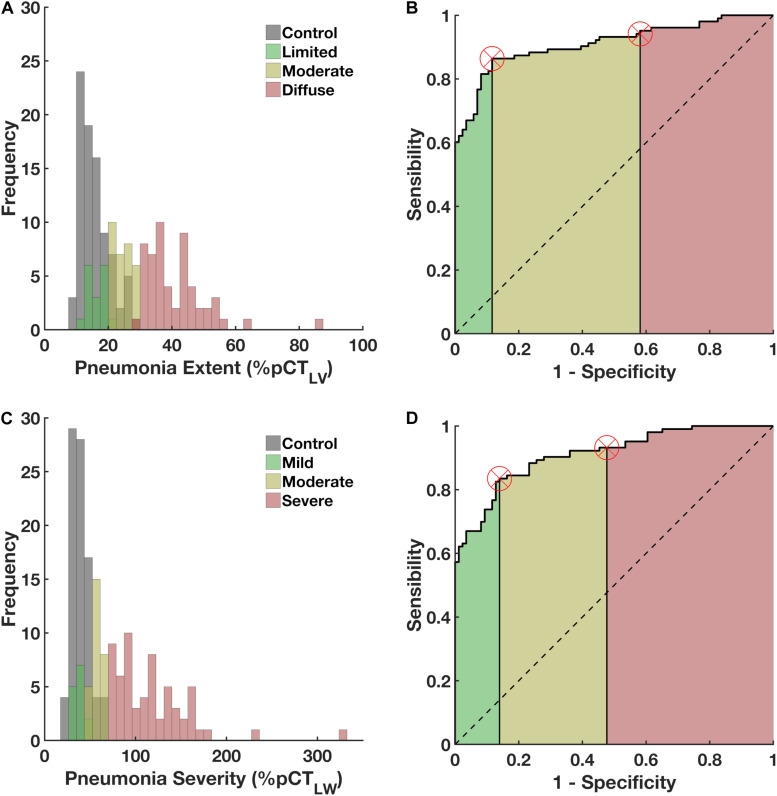
Left panels **(A,C)** Histograms of the frequency of occurrence of pneumonia extent and severity in patients with COVID-19 (green, yellow and red colored) and the volume and weight associated to parenchymal opacities in controls (gray). Figure 3 right panels **(B,D)** ROC curves, with the areas under the curves (AUCs) hatched in green, yellow and red. Vertical lines mark the normality cut-off (equivalent to 20% of the predicted total lung volume represented by pulmonary opacities and 50% of the predicted lung weight represented by pulmonary opacities) and the *Z* score of 3 (equivalent to 30% and 60%, respectively, and used to classify COVID-19 patients as having diffuse and severe pneumonia). Values falling between the normality cut-off and the *Z* score of 3 represent moderate pulmonary involvement. The use of parenchymal opacity adjusted to the predicted lung volume as an indicator of pneumonia extent presented 0.80 sensitivity, 0.86 specificity, an AUC of 0.91, accuracy of 0.82, 0.90 positive predictive value, 0.73 negative predictive value, *F* score of 0.85, and Matthews correlation coefficient of 0.65. Pneumonia severity presented 0.86 sensitivity, 0.88 specificity, an AUC of 0.91, accuracy of 0.87, 0.90 positive predictive value, 0.84 negative predictive value, *F* score of 0.88, and Matthews correlation coefficient of 0.75.

The ROC curve indicated that the optimal threshold for pneumonia extent was 20% (sensitivity = 0.80, specificity = 0.85, AUC = 0.91 95%CI 0.88-0.93, accuracy = 0.82, *F* score = 0.85, Matthews’ correlation coefficient = 0.65; Figures 3A,B). Accordingly, COVID-19 patients were classified based as with diffuse (≥*Z*-score of 3, in 55 patients), moderate (from 20% to *Z*-score of 3, in 31 patients) and limited pneumonia (<20%, in 17 patients).

The optimal threshold for pneumonia severity was 50% (sensitivity = 0.86, specificity = 0.88, AUC = 0.91 95%CI 0.89–0.92, accuracy = 0.87, *F*-score = 0.88, Matthews’ correlation coefficient = 0.75, Figures 3C,D). COVID-19 patients were classified as with severe PI (≥*Z-*score of 3, in 61 patients), moderate (from 20% to *Z*-score of 3, in 28 patients) and mild pneumonia severity (<20%, in 14 patients).

**FIGURE 4:**
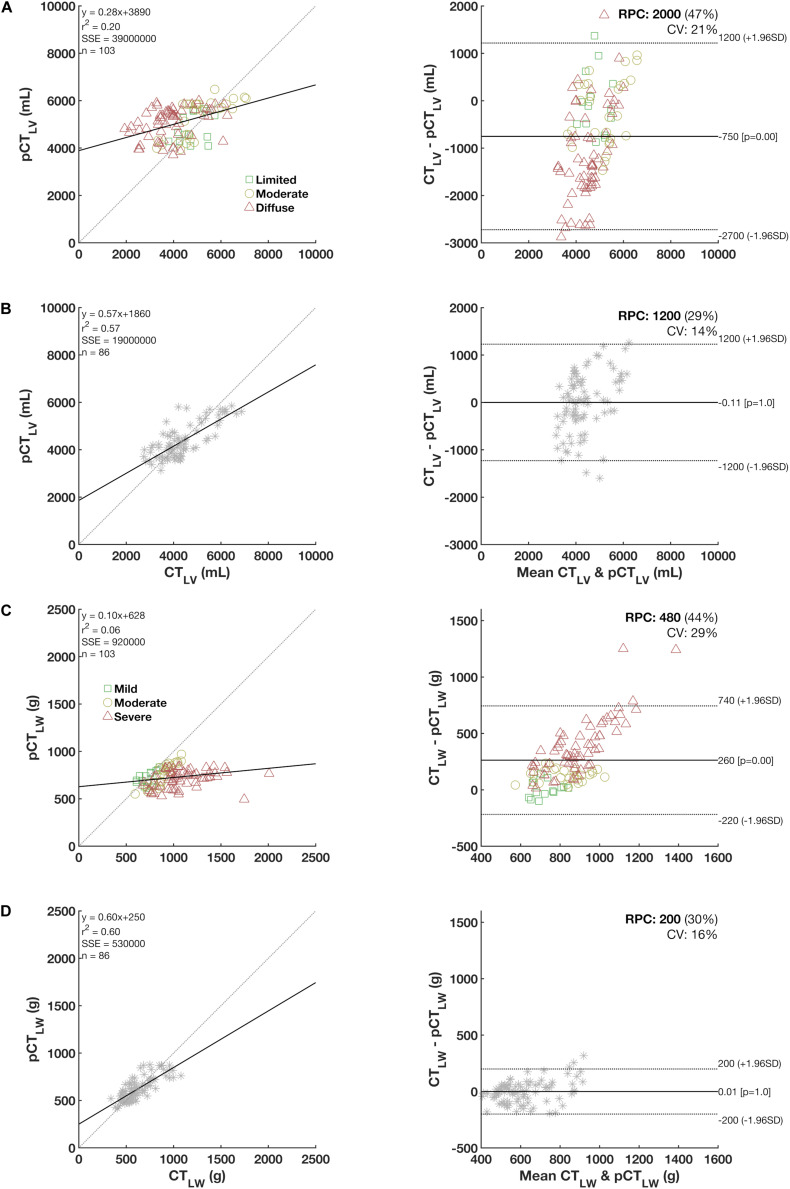
Left panels: The relationship between predicted (pCT_LV_) and measured lung volumes (CT_LW_) and weight in COVID-19 patients **(A,C)** and controls **(B,D)** with each respective Bland-Altman bias plot (right panels). Note that in controls, no significant bias is observed whereas the bias tended to increase in more diffuse and severe COVID-19 pneumonia patients (colored in red).

Figure 4 depicts the relationship and the Bland-Altman bias plot between CT_LV_ and pCT_LV_ in COVID-19 patients and controls (Figures 4A,B) and between CT_LW_ and pCT_LW_ (Figures 4C,D). Note that the agreements between measured and predicted values were quite reduced in patients with more diffuse and severe COVID-19 pneumonia, whereas almost no bias was observed in controls.

**FIGURE 5:**
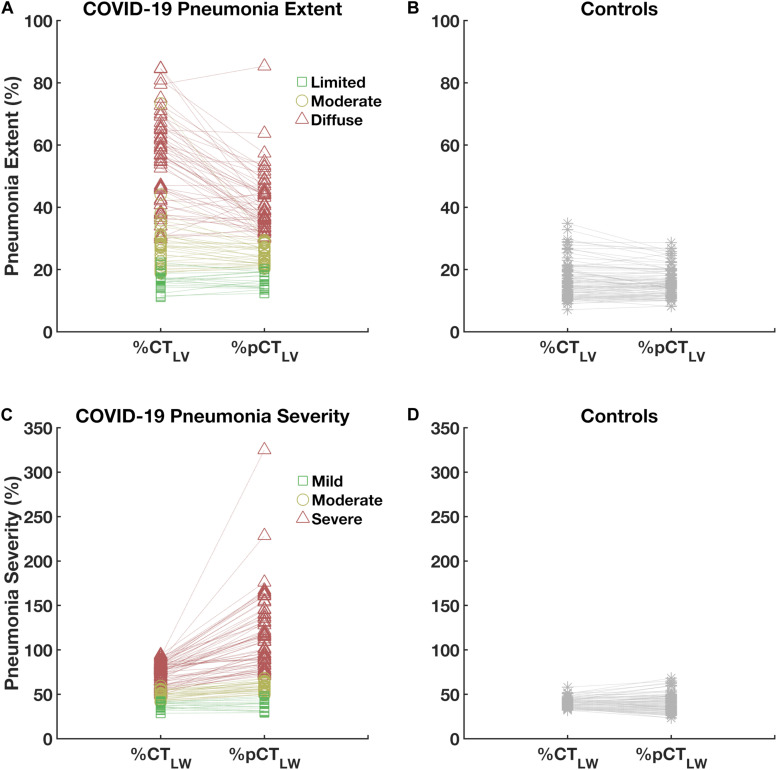
Paired plot of pneumonia extent (upper panels, **A,B**) and severity (lower panels, **C,D**) expressed as percentages of computed tomography-estimated lung volume (%CTLV) and weight (%CTLW) and predicted computed tomography-estimated lung volume (%pCT_LV_) and weight (%pCT_LW_) in patients (left panels, **A,C**) and controls (right panels, **B,D**). Note that the difference in the estimation of pneumonia extent and severity from non-normalized and normalized are higher in diffuse and severe COVID-19 cases (triangles).

The COVID-19 pneumonia extent was significantly lesser when adjusted to the pCT_*L*__*V*_ than when adjusted to the CT_*L*__*V*_ (40.8 ± 19.9% vs. 32.1 ± 12.6%, *P* < 0.001; Figure 5A). Noteworthy, pneumonia severity was significantly higher when adjusted to the pCT_LW_ (62.5 ± 17.8% vs. 90.3 ± 47%, *P* < 0.001; Figure 5C) specially in moderate and severe COVID-19 patients. No significant variations were observed in the control group (Figures 5B,D). The greatest difference these indicators was observed in patients classified as with more severe disease (Figure 5).

**FIGURE 6:**
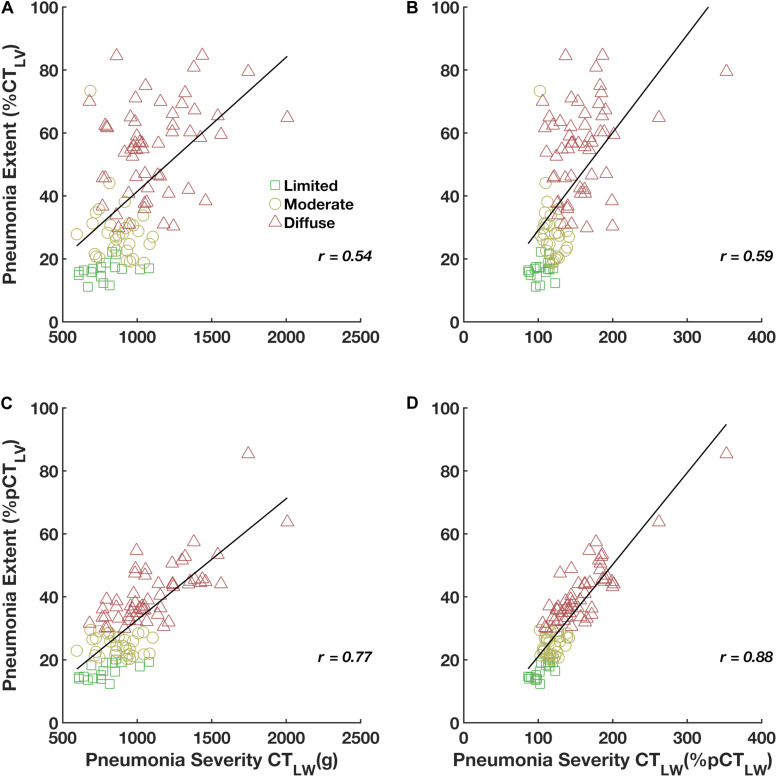
Pearson’s correlations of pneumonia extent expressed as%CT_LV_ with the CT_LW_ in grams **(A)** and expressed as%pCT_LW_ (pneumonia severity, **B**), and of pneumonia extent expressed as%pCT_LV_ with the CT_LW_ in grams **(C)** and expressed as%pCT_LW_ (pneumonia severity, **D**).

The relationship between pneumonia extent and severity was improved when both were adjusted to pCT_LV_ and pCT_LW_, respectively (Figure 6D). The Pearson correlation coefficient between pneumonia extent expressed as%CT_LV_ and the pneumonia severity expressed in grams was just moderate (*r* = 0.54, Figure 6A), whereas that between the pneumonia extent expressed as a percentage of the pCT_LV_ and the pneumonia severity expressed as a percentage of the pCT_LW_ was very strong (*r* = 0.88; Figure 6D). In addition, a considerable reduction in the overall dispersion was observed.

Relative to patients with moderate and limited pneumonia extent and moderate and mild pneumonia severity, patients with COVID-19 diffuse and severe pneumonia were older and had higher CRP concentrations at admission. Additionally, a positive and moderate correlation between pneumonia severity (*r* = 0.46) and age and CRP concentration (*r* = 0.44) was observed. Moreover, COVID-19 most diffuse and severe pneumonia required ICU stay and ventilatory support, presenting higher intra-hospital mortality. Moreover, they exhibited significantly reduced CT_LV_s and increased CT_LW_s. As expected, the volume of GGO, CP/LO, and consolidation significantly increased in patients with diffuse and severe pneumonia extent and severity (Tables 1, 2).

**TABLE 1:** Demographic, anthropometric, clinical, and computed tomography data on patients classified with limited, moderate and diffuse COVID-19 pneumonia extent and controls.

	COVID-19 pneumonia extent *N* = 103			Controls *N* = 86	*P*-values
	Diffuse *N* = 55	Moderate *N* = 31	Limited *N* = 17		
**Demographic and anthropometrics**					
Age (y)	66 ± 14^b^	59 ± 15	50 ± 15	60 ± 20	0.01^b^
Gender (female)	16	11	7	62	–
Body height (m)	1.7 ± 0.1^d^	1.7 ± 0.1^d^	1.7 ± 0.1^d^	1.6 ± 0.1	<0.001^d^
Body weight (kg)	79.8 ± 14.3^d^	83.4 ± 18.8^d^	78.9 ± 20.3^d^	66.6 ± 13.3	<0.01^d^
BMI (kg/m^2^)	28.2 ± 4.4^d^	28.1 ± 5.3	26.4 ± 4.8	25.7 ± 4.3	0.01^d^
**Laboratory data**					
White blood count (×10^3^/μL)	6.5 ± 3.1	5.5 ± 3.3	5.3 ± 1.2		
Lymphocytes count (×10^3^/μL)	1.2 ± 1.3	1.1 ± 0.4	1.2 ± 0.5		
Lactate dehydrogenase (U/L)	312.8 ± 128.4	300.7 ± 137.5	227.6 ± 99.1		
CRP (mg/L)	54.2^a,b^ ± 63.3	6.7 ± 8.7	5.1 ± 5.8		<0.002^a,b^
GOT (U/L)	38.7 ± 24.0	36.9 ± 21.8	22.1 ± 36.9		
GPT (U/L)	36.5 ± 22.9	37.9 ± 31.4	33.5 ± 33.5		
Creatinine (mg/dL)	0.98 ± 0.48	1.3 ± 1.7	0.84 ± 0.26		
**Clinical outcomes**					
Symptoms onset (days)	8.8 ± 5.6	6.7 ± 5.4	8.0 ± 7.8	–	
ICU (%)	17	11	4	–	
i-MV (%)	9	6	2	–	
In-hospital mortality (%)	9	3	0	–	
**CT data**					
CT_LV_ (mL)	3,916 ± 990^a,b^	4,639 ± 1,041	5,199 ± 869	4,350 ± 958	<0.006^a,b^
pCT_LV_ (mL)	5,052 ± 654^d^	5,156 ± 763^d^	5,133 ± 686^d^	4,350 ± 725	<0.001^d^
CT_LW_ (g)	1,113 ± 260^a,b,d^	872 ± 127^c,d^	785 ± 132^d^	621 ± 161	<0.008^a,b,c,d^
pCT_LW_ (g)	706 ± 92^d^	738 ± 114^d^	752 ± 79^d^	621 ± 124	<0.001^d^
Pneumonia extent (%CT_LV_)	55 ± 14^a,b,d^	29 ± 10^c,d^	17 ± 3^d^	16 ± 6	<0.001^a,b,c,d^
Pneumonia extent (%pCT_LV_)	41 ± 10^a,b,d^	25 ± 3^c,d^	16 ± 3	16 ± 5	<0.001^a,b,c,d,^
GGO (mL)	1,363 ± 424^a,b,d^	906 ± 151^c,d^	571 ± 143	507 ± 230	<0.001^a,b,c,d^
CP/LO (mL)	527 ± 266^a,b,d^	281 ± 98^d^	219 ± 86	136 ± 36	<0.001^a,b,d^
Consolidation (mL)	192 ± 157^a,b,d^	72 ± 25	54 ± 19	43 ± 16	<0.001^a,b,d^

**^*a*^Severe vs. moderate, ^*b*^Severe vs. mild, ^*c*^Moderate vs. mild, ^d^COVID-19 vs. controls. Data are shown as means ± standard deviations. BMI, body mass index; CRP, C-reactive protein; GOT, glutamic oxaloacetic transaminase; GPT, glutamic pyruvic transaminase; ICU, intensive care unit; i-MV, invasive mechanical ventilation, CT, computed tomography; CT_LV_, computed tomography–calculated total lung volume; pCT_LV_, predicted computed tomography–estimated total lung volume; CT_LW_, computed tomography–calculated total lung weight; pCT_LW_, predicted computed tomography–estimated total lung weight; GGO, volume related to ground-glass opacities; CP/LO, volume related to crazy paving and linear opacities; consolidation, volume related to consolidation.**

**TABLE 2:** Demographic, anthropometric, clinical, and computed tomography data on patients classified with limited, moderate and diffuse COVID-19 pneumonia severity and controls.

	COVID-19 pneumonia severity *N* = 103			Controls *N* = 86	*P*-values
	Severe *N* = 61	Moderate *N* = 28	Mild *N* = 14		
**Demographic and anthropometrics**					
Age (y)	67 ± 13^a,b^	55 ± 16	48 ± 15	60 ± 20	0.018^a^ 0.001^b^
Gender (male/female)	17/44	9/19	8/6	24/62	–
Body height (m)	1.7 ± 0.1^d^	1.7 ± 0.1^d^	1.7 ± 0.05^d^	1.6 ± 0.1	<0.009^d^
Body weight (kg)	81 ± 14.4^d^	85 ± 21^d^	73 ± 15.7	66.6 ± 13.3	<0.003^d^
BMI (kg/m^2^)	28.4 ± 4.4^d^	28 ± 5.4	25.4 ± 4.5	25.7 ± 4.3	0.003^d^
**Laboratory data**					
White blood count (× 10^3^/μL)	6.6 ± 3.0	5.5 ± 3.3	4.8 ± 1.1	–	
Lymphocytes count (× 10^3^/μL)	1.2 ± 1.3	1.2 ± 0.4	1.2 ± 0.6	–	
Lactate dehydrogenase (U/L)	315.3 ± 126.5^b^	304.7 ± 139.1	194.4 ± 59.5	–	0.028^b^
CRP (mg/L)	51.0 ± 61.2^a,b^	6.4 ± 8.4	3.8 ± 5.1	–	<0.006^a,b^
GOT (U/L)	39.8 ± 22.7	35.6 ± 22.1	34.0 ± 25.0	–	
GPT (U/L)	38.7 ± 23.4	34.5 ± 30.2	37.4 ± 37.6	–	
Creatinine (mg/dL)	1.0 ± 0.5	1.3 ± 1.7	0.8 ± 0.3	–	
**Clinical outcomes**					
Symptoms onset (days)	8.7 ± 5.5	7.2 ± 6.6	8.0 ± 7.8	–	–
ICU (%)	34	28	21	–	–
i-MV (%)	20	18	8	–	–
In-hospital mortality (%)	10	0	0	–	–
**CT data**					
CT_LV_ (mL)	3,909 ± 1,008^a,b,d^	5,060 ± 993^d^	4,816 ± 553	4,350 ± 958	<0.04^a,b,d^
pCT_LV_ (mL)	5,072 ± 627^d^	5,267 ± 800^d^	4,863 ± 588	4,350 ± 725	<0.001^d^
CT_LW_ (g)	1,089 ± 260^a,b,d^	891 ± 116^d^	729 ± 77	621 ± 161	<0.001^a,b,d^
pCT_LW_ (g)	706 ± 90^d^	761 ± 114^d^	720 ± 68^d^	621 ± 124	<0.013^d^
Pneumonia severity (%CT_LW_)	75 ± 12^a,b,d^	49 ± 5^c,d^	37 ± 4	39 ± 5	<0.001^a,b,c,d^
Pneumonia severity (%pCT_LW_)	117 ± 42^a,b,d^	58 ± 5^d^	38 ± 6	39 ± 10	<0.007^a,b,d^
GGO (mL)	1,314 ± 422^a,b,d^	891 ± 190^d^	545 ± 136	507 ± 230	<0.001^a,b,d^ 0.003^c^
CP/LO (mL)	517 ± 251^a,b,d^	264 ± 88^d^	181 ± 57	136 ± 36	<0.001^a,b,d^
Consolidation (mL)	183 ± 150^a,b,d^	68 ± 21	45 ± 12	43 ± 16	<0.001^a,b,d^

**^*a*^Severe vs. moderate, ^*b*^Severe vs. mild, ^*c*^Moderate vs. mild, ^d^COVID-19 vs. controls. Data are shown as means ± standard deviations. BMI, body mass index; CRP, C-reactive protein; GOT, glutamic oxaloacetic transaminase; GPT, glutamic pyruvic transaminase; ICU, intensive care unit; i-MV, invasive mechanical ventilation, CT, computed tomography; CT_LV_, computed tomography–calculated total lung volume; pCT_LV_, predicted computed tomography–estimated total lung volume; CT_LW_, computed tomography–calculated total lung weight; pCT_LW_, predicted computed tomography–estimated total lung weight; GGO, volume related to ground-glass opacities; CP/LO, volume related to crazy paving and linear opacities; consolidation, volume related to consolidation.**

## Discussion

In COVID-19 pneumonia, lung terminal structures, such as the interlobular septum and alveolar wall, can be involved and cause extensive edema and lymphocyte infiltration in the lung interstitium (Kim et al., 2002). Although early alveolar exudation is not prominent, the disease progresses rapidly (Li et al., 2020). Thus, the quantification of COVID-19 pneumonia extent and severity from chest CT images might be of clinical interest for risk stratification and can have some prognostic value (Colombi et al., 2020; Li et al., 2020).

In the present study, we propose a methodology to estimate COVID-19 pneumonia extent and severity and verified the possible associations between those indicators with clinical and radiological outcomes.

Our results suggested that COVID-19 pneumonia extent can be less biased calculated as the ratio between the volume of lung parenchyma opacities from chest CT images adjusted to the predicted CT_LV_ (Figure 4). Accordingly, an increase in the volume of abnormal opacities in the lung parenchyma would indicate an increase in the extension of pneumonia without the bias associated with the fact that larger pneumonia would also reduce the total lung volume (Patroniti et al., 2005).

As complementary information, pneumonia severity was assessed by calculating the ratio between the weight of abnormal parenchymal opacities and the predicted CT_LW_. In fact, an interesting application of the CT_LW_ estimation was the potential to distinguish pulmonary opacification in the context of inflammatory alveolar flooding from compression atelectasis (Reske et al., 2011). Therefore, patients with pulmonary opacification on CT images but with normal lung weight likely present more atelectasis due to hypoventilation, the use of anesthetics, and high inspired oxygen fractions. Alternatively, increased lung weight suggests consolidation due to significant lung injury, for example, hemorrhage or edema caused by capillary leakage (Patroniti et al., 2005; Reske et al., 2011).

The differential diagnostic ability of the proposed method to determine pneumonia extent and severity in COVID-19 pneumonia was assessed by the ROC analysis with almost the same AUC, sensitivity, specificity and accuracy (Figure 3). Thus, 55 COVID-19 patients were classified as having diffuse pneumonia extent and 61 as presenting severe pneumonia. Those patients were older and seemed to be more toxemic (higher CRP concentrations at admission) and more frequently required ICU and ventilatory support and presented higher intra-hospital mortality. Moreover, COVID-19 patients with diffuse and severe pneumonia also presented significant volumes of GGO, CP/LO and consolidation (Tables 1, 2).

Patients with diffuse and severe COVID-19 pneumonia almost certainly present denser lung parenchyma, likely because pulmonary opacifications represent a more exuberant inflammatory component with more alveolar flooding and cell infiltration than seen with atelectasis or ventilatory defects.

Interestingly, pneumonia extent expressed as%CT_LV_ was only moderately associated with the CT_LW_ while pneumonia extent expressed as%pCT_LV_ correlated very strongly with the CT_LW_ adjusted to the pCT_LW_ (pneumonia severity), with a considerable reduction in the overall dispersion. These results emphasize the importance of adjustment to predicted values to achieve the unbiased use of pneumonia extent as an indicator of disease severity in patients with COVID-19 pneumonia (Figure 6).

Several predictive equations have been used for the normalization of pulmonary function data (Roberts et al., 1991; Roca et al., 1998). However, few reference values for CT_LV_ and CT_LW_ in healthy subjects have been reported (Reske et al., 2011; Cressoni et al., 2013). Most predictive equations used for pulmonary function data are obtained from persons of both sexes and of various ages, heights, and ethnicities. However, many of them used seated individuals, which clearly increases all static lung volumes compared with those obtained while subjects are supine, the most common position for CT examination (Reske et al., 2011; Cressoni et al., 2013). That was the main motivation for the assessment of such predictive equations in our control group.

In the present study, age ranged widely (from 22 to 81 years) in the control group, but female sex predominated. Despite this limitation, our predictive equations yielded acceptable adjusted *R*^2^ values for CT_LV_ and CT_LW_ prediction (Figure 4) and are in accordance with the literature (Whimster and Macfarlane, 1974; Gattinoni et al., 2006; Puybasset et al., 2000b; Reske et al., 2011; Cressoni et al., 2013). Further studies with more heterogeneous populations would be of great value for the development of new and more representative predictive equations.

### Study Limitation

One important limitation of the methodology proposed here is the need for data on subjects’ height, which is not always included in clinical records. We recommend the routine annotation of subjects’ height, which would be of great benefit for further studies and data normalization. However, it is important to note that the impact of the normalization by predicted CT_LV_ or CT_LW_ was always greater in estimating the extent, but not the severity, of COVID-19 pneumonia (Figure 6). This can be explained, albeit partially, by the negative contribution of age in lung weight estimation. In fact, it is noted that patients with more diffuse and severe COVID-19 pneumonia were older than the others, including controls.

In summary, the method proposed in the present study might be of clinical interest for the determination of COVID-19 pneumonia extent and severity and might be useful for clinical and radiological patient stratification. Further studies are necessary to assess the validation of proposed pneumonia extent and severity indicators at the clinical scenario. Indeed, the association between the extent and severity of COVID-19 pneumonia with the clinical outcomes or even inflammatory markers still need to be better assessed.

## Data Availability Statement

The original contributions presented in the study are included in the article/supplementary material, further inquiries can be directed to the corresponding author/s.

## Ethics Statement

The studies involving human participants were reviewed and approved by the Hospital Copa Star and Barra D’Or, Rio de Janeiro Brazil and Hospital de Santo António, CHUP, Porto, Portugal. Written informed consent for participation was not required for this study in accordance with the national legislation and the institutional requirements.

## Author Contributions

AC: image processing and analysis of results, statistical evaluation, theoretical development of the neural network and the computation method of voxel to voxel analysis, writing of the text, and submission of the article. AG: image processing and segmentation, statistical evaluation, neural network implementation. TG: image segmentation, capture and organization of clinical data, and draft review. GM: determination of image regions of interest, capture and organization of clinical data, and draft review. VC: determination of image regions of interest and image segmentation. FB: results discussion and draft review. RR: capture and organization of clinical data, results discussion, and draft review. JP: capture and organization of clinical data. WS: determination of image regions of interest and draft review. WZ: draft and final review. MF: capture and organization of clinical data and draft review. All authors approved the final version of the manuscript.

## Conflict of Interest

The authors declare that the research was conducted in the absence of any commercial or financial relationships that could be construed as a potential conflict of interest.

## Abbreviations

ANN: Artificial neural network
AUC: Area under the ROC curve
COVID-19: Coronavirus disease 2019
CP/LO: Crazy paving/linear opacities
CT: Computed tomography
CTLV: Computed tomography–estimated lung volume
CTLW: Computed tomography–estimated lung weight
GGO: Ground-grass opacities
pCTLV: Predicted computed tomography–estimated lung volume
pCTLW: Predicted computed tomography–estimated lung weight
PO: Pulmonary opacities
ROC: Receiver operating characteristic
ROI: Region of interest.

## References

[B1] Brismar B. Hedenstierna G. Lundquist H. Strandberg Å Svensson L. Tokics L. (1985). Pulmonary densities during anesthesia with muscular relaxation–a proposal of atelectasis. *Anesthesiology* 62 422–428. 10.1097/00000542-198504000-00009 3885791

[B2] Carvalho A. R. S. Guimarães A. Werberich G. M. de Castro S. N. Pinto J. S. F. Schmitt W. R. (2020). COVID-19 chest computed tomography to stratify severity and disease extension by artificial neural network computer-aided diagnosis. *Front. Med.* 7:577609. 10.3389/fmed.2020.577609 33344471 PMC7746855

[B3] Colombi D. Bodini F. C. Petrini M. Maffi G. Morelli N. Milanese G. (2020). Well-aerated lung on admitting chest CT to predict adverse outcome in COVID-19 pneumonia. *Radiology* 296 E86–E96. 10.1148/radiol.2020201433 32301647 PMC7233411

[B4] Cressoni M. Gallazzi E. Chiurazzi C. Marino A. Brioni M. Menga F. (2013). Limits of normality of quantitative thoracic CT analysis. *Crit. Care* 17:R93. 10.1186/cc12738 23706034 PMC4057220

[B5] Fedorov A. Beichel R. Kalpathy-Cramer J. Finet J. Fillion-Robin J.-C. Pujol S. (2012). 3D Slicer as an image computing platform for the quantitative imaging network. *Magn. Reson. Imaging* 30 1323–1341. 10.1016/j.mri.2012.05.001 22770690 PMC3466397

[B6] Gattinoni L. Caironi P. Cressoni M. Chiumello D. Ranieri V. M. Quintel M. (2006). Lung recruitment in patients with the acute respiratory distress syndrome. *N. Engl. J. Med.* 354 1775–1786. 10.1056/nejmoa052052 16641394

[B7] Gattinoni L. Coppola S. Cressoni M. Busana M. Rossi S. Chiumello D. (2020). Covid-19 does not lead to a “Typical” acute respiratory distress syndrome. *Am. J. Respir. Crit. Care Med.* 201 1299–1300. 10.1164/rccm.202003-0817le 32228035 PMC7233352

[B8] Hope M. D. Raptis C. A. Shah A. Hammer M. M. Henry T. S. (2020). A role for CT in COVID-19? What data really tell us so far. *Lancet* 395 1189–1190. 10.1016/s0140-6736(20)30728-5 PMC7195087 32224299

[B9] Kim E. A. Lee K. S. Primack S. L. Yoon H. K. Byun H. S. Kim T. S. (2002). Viral pneumonias in adults: radiologic and pathologic findings1. *Radiographics* 22 S137–S149. 10.1148/radiographics.22.suppl_1.g02oc15s137 12376607

[B10] Li K. Fang Y. Li W. Pan C. Qin P. Zhong Y. (2020). CT image visual quantitative evaluation and clinical classification of coronavirus disease (COVID-19). *Eur. Radiol.* 30 4407–4416. 10.1007/s00330-020-06817-6 32215691 PMC7095246

[B11] Mull R. T. (1984). Mass estimates by computed tomography: physical density from CT numbers. *AJR* 143 1101–1104. 10.2214/ajr.143.5.1101 6333158

[B12] Patroniti N. Bellani G. Maggioni E. Manfio A. Marcora B. Pesenti A. (2005). Measurement of pulmonary edema in patients with acute respiratory distress syndrome. *Crit. Care Med.* 33 2547–2554. 10.1097/01.ccm.0000186747.43540.25 16276179

[B13] Protti A. Iapichino G. E. Milesi M. Melis V. Pugni P. Comini B. (2014). Validation of computed tomography for measuring lung weight. *Intensive Care Med. Exp.* 2:31. 10.1186/s40635-014-0031-0 26266928 PMC4512984

[B14] Puybasset L. Cluzel P. Gusman P. Grenier P. Preteux F. Rouby J.-J. (2000a). Regional distribution of gas and tissue in acute respiratory distress syndrome. I. Consequences for lung morphology. CT Scan ARDS Study Group. *Intensive Care Med.* 26 857–869. 10.1007/s001340051274 10990099

[B15] Puybasset L. Gusman P. Muller J. C. Cluzel P. Coriat P. Rouby J.-J. (2000b). Regional distribution of gas and tissue in acute respiratory distress syndrome. III. Consequences for the effects of positive end-expiratory pressure. CT scan ARDS study group. Adult respiratory distress syndrome. *Intensive Care Med.* 26 1215–1227. 10.1007/s001340051340 11089745

[B16] Rello J. Storti E. Belliato M. Serrano R. (2020). Clinical phenotypes of SARS-CoV-2: implications for clinicians and researchers. *Eur. Respir. J.* 12:2001028. 10.1183/13993003.01028-2020 32341111 PMC7236837

[B17] Reske A. W. Reske A. P. Heine T. Spieth P. M. Rau A. Seiwerts M. (2011). Computed tomographic assessment of lung weights in trauma patients with early posttraumatic lung dysfunction. *Critical Care* 15:R71. 10.1186/cc10060 21352529 PMC3222004

[B18] Roberts C. M. MacRae K. D. Winning A. J. Adams L. Seed W. A. (1991). Reference values and prediction equations for normal lung function in a non-smoking white urban population. *Thorax* 46 643–650. 10.1136/thx.46.9.643 1948793 PMC463354

[B19] Roca J. Burgos F. Barberà J. A. Sunyer J. Rodriguez-Roisin R. Castellsagué J. (1998). Prediction equations for plethysmographic lung volumes. *Respir. Med.* 92 454–460. 10.1016/s0954-6111(98)90291-8 9692105

[B20] Staring M. Bakker M. E. Stolk J. Shamonin D. P. Reiber J. H. C. Stoel B. C. (2016). Towards local progression estimation of pulmonary emphysema using CT. *Med. Phys.* 41 21905–21914. 10.1118/1.4851535 24506626

[B21] Whimster W. F. Macfarlane A. J. (1974). Normal lung weights in a white population. *Am. Rev. Respir. Dis.* 110:478. 10.1164/arrd.1974.110.4.478 4414844

[B22] Yang S. Jiang L. Cao Z. Wang L. Cao J. Feng R. (2020). Deep learning for detecting corona virus disease 2019 (COVID-19) on high-resolution computed tomography: a pilot study. *Ann. Transl. Med.* 8:450. 10.21037/atm.2020.03.132 PMC7210135 32395494

